# Degradability of chitosan micro/nanoparticles for pulmonary drug delivery

**DOI:** 10.1016/j.heliyon.2019.e01684

**Published:** 2019-05-15

**Authors:** Nazrul Islam, Isra Dmour, Mutasem O Taha

**Affiliations:** a Pharmacy Discipline, School of Clinical Sciences, Faculty of Health, Queensland University of Technology (QUT), Brisbane, QLD 4000, Australia; b Institute of Health and Biomedical Innovation, QUT, 60 Musk Avenue, Kelvin Grove, Brisbane, QLD 4059, Australia; c Faculty of Pharmacy and Medical Sciences, Al-Ahliyya Amman University, Amman, Jordan; d Department of Pharmaceutical Sciences, Faculty of Pharmacy, University of Jordan, Amman, 11942 Jordan

**Keywords:** Analytical chemistry, Bioengineering, Biogeoscience, Biomedical engineering, Cancer research, Infectious disease, Materials chemistry, Nanotechnology, Pharmaceutical chemistry, Physical chemistry

## Abstract

Chitosan, a natural carbohydrate polymer, has long been investigated for drug delivery and medical applications due to its biodegradability, biocompatibility and low toxicity. The micro/nanoparticulate forms of chitosan are reported to enhance the efficiency of drug delivery with better physicochemical properties including improved solubility and bioavailability. This polymer is known to be biodegradable and biocompatible; however, crosslinked chitosan particles may not be biodegradable. Crosslinkers (e.g., tripolyphosphate and glutaraldehyde) are needed for efficient micro/nanoparticle formation, but it is not clear whether the resultant particles are biodegradable or able to release the encapsulated drug fully. To date, no studies have conclusively demonstrated the complete biodegradation or elimination of chitosan nanoparticles *in vivo*. Herein we review the synthesis and degradation mechanisms of chitosan micro/nanoparticles frequently used in drug delivery especially in pulmonary drug delivery to understand whether these nanoparticles are biodegradable.

## 1 Introduction

Polymer micro/nanoparticles prepared using different techniques have been extensively studied for drug delivery systems [1, 2, 3] due to their biodegradability and biocompatibility properties. The term ‘biodegradability’ has been defined by IUPAC as the breakdown of polymers due to cellular or *in vivo* biological actions [4]while ‘biocompatibility’ refers to the property of materials that act favourably with a biological system by not producing a toxic, injurious or immunogenic response. These two phrases are frequently used in developing polymer-based medical devices and micro/nanoparticles for drug delivery without sufficient supporting studies of the degradation of polymer nanoparticles, identification of the degradation products and their subsequent effects in biological systems.

Many polymers (especially the chitosan) and their nano/microparticles, commonly referred to as biodegradable/biocompatible, have been investigated for drug delivery and other medical purposes; however, the mechanism of their degradation has not been fully elucidated in biological systems. Evidence for their *in vitro* or *in vivo* degradation is very limited or conflicting. The FDA has approved some polymers for drug delivery and certain medical applications [5] because their degradation products are deemed to be biocompatible and eliminated from the body. However, once these polymers have formed nanoparticles by crosslinking (either by covalent, ionic or other bonds), it is not known whether those nano/microparticles are entirely biodegradable or biocompatible.

Chitosan, a natural polymer known as being biocompatible and biodegradable has been studied frequently for the delivery of various drugs, vaccines, genes and chemotherapeutic agents [6, 7, 8, 9] Various types of chitosan-based carriers have been investigated for pulmonary delivery of various drugs such as isoniazide [10], ciprofloxacin [11], gentamicin [12], heparin [13], etc. have been investigated. The applications of chitosan-based nanoparticles/nanobiocomposites in wound dressing, pulmonary drug delivery, tissue engineering, and biosensors are increasing [8, 14, 15]with a firm belief that these materials are biodegradable and biocompatible without comprehensive supporting data. Chitosan micro/nanoparticles have been extensively investigated as a carrier for lung drug delivery [8, 16, 17, 18, 19, 20]. Very recently, we have demonstrated that the chitosan with a high degree of deacetylation (92%) and its nanoparticles crosslinked with glutaraldehyde are not degradable in lysozyme solution (0.2 mg/mL) that mimic the enzyme concentration in lung fluid [21]. To date, no studies have conclusively demonstrated the complete biodegradation or elimination of chitosan nanoparticles prepared by different techniques especially for delivering drugs into lungs from where removal of nanoparticles or degraded products is not known. This mini-review examines contemporary research on the biodegradability of crosslinked chitosan micro/nanoparticles and their subsequent impact on drug delivery and pathways for removal of the polymers or degradation products. Additionally, the chemistry of chitosan and chitosan crosslinked with glutaraldehyde and sodium tripolyphosphate (TPP) is examined to understand the factors that may control the degradability of chitosan micro/nanoparticles in *in-vitro*/*in-vivo* systems.

## 2 Main text

### 2.1 Chemistry of chitosan, cross-linked chitosan with TPP and glutaraldehyde

#### 2.1.1 Chemistry of chitosan

**Fig. 1:**
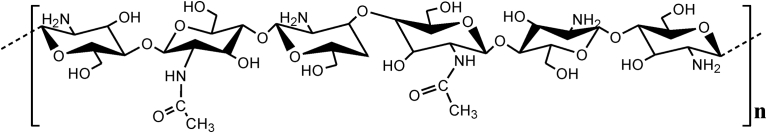
Chemical structure of Chitosan showing residual N-acetyl groups from the parent chitin.

Chitosan is a gel- and film-forming linear polysaccharide that can bind metal ions and organic compounds, e.g., in water filtration [22]. Chemically, chitosan is a β-(1→4)-2-amino-2-deoxy-D-glucan obtained by partial *N*-deacetylation of chitin (Fig. 1). It consists of D-glucosamine and occasional *N*-acetyl-D-glucosamine units that are bonded via β(1→4) linkages. The degree of deacetylation affects many of its chemical and physical properties. As a primary aliphatic amine, chitosan can be protonated by selected acids, with the p*K*a of the chitosan amine being 6.2–6.5 [23]. The following salts, among others, are water-soluble: formate, acetate, lactate, malate, citrate, glyoxylate, pyruvate, glycolate, and ascorbate [24]. These glucosamine residues carry positive charges at slightly acidic pH values, making chitosans polycationic biopolymers which can easily interact with polyanionic molecules such as many proteins, DNA, or phospholipids. Also, the presence of both hydroxy and amino groups provides many options for chemical modification [25]. These modifications offer new polymeric materials with varying physicochemical and biopharmaceutical properties, e.g., solubility, adsorption, pH sensitivity, and thermoresponsive properties with diverse functionalities [26, 27]. Chemical modifications of chitosan included carboxymethylation, thiolation, succinylation, grafting, and copolymerization, among others [28]. The chitosan derivatives have been synthesised to have their improved solubility, enhance cellular uptake, less toxicity, and encapsulate different types of drugs with sustained release profile. It can be pointed out that nanoparticles of chitosan derivatives have generally shown acceptable cytotoxicity [29], which was evaluated by using different techniques. For example, the toxicities of chitosan, quaternised chitosan, and chitosan phthalate, amphiphilic chitosan nanoparticle were assessed using MTT assay [30, 31, 32]. The cytotoxicity of thiolated chitosan was investigated using Red blood cells lysis [33] while Calu-3 cells, a model of human respiratory function was used to evaluate trimethyl chitosan [34].

Chitosan properties such as molecular weight, degree of acetylation (DA) and degree of crystallinity, highly determine its mechanical and biological properties [35]. It is well established that chitosan with a low DA tended to be degraded more rapidly. Chitosan degradation is dependent on variations in the distribution of acetamide groups in the chitosan molecule. This occurs due to differences in deacetylation conditions which influences the viscosity of the chitosan solution by changing the inter- or intra-molecular repulsion forces. However, it is impossible to estimate the biodegradation rate of chitosan using the DA alone [36]. The molecular weight of chitosan may change from 300 to over 1000 kD which affects many of its properties like viscosity. Therefore, the greater the molecular weight is, the chitosan membranes tend to be more viscous. Due to its high molecular weight and its lineal nonbranched structure, chitosan is a strong viscosity-building agent in acid mediums and behaves as a pseudoplastic material, where viscosity depends on agitation. There is a direct association between molecular weight and DA [37].

#### 2.1.2 Chemistry of chitosan crosslinked with TPP

**Fig. 2:**
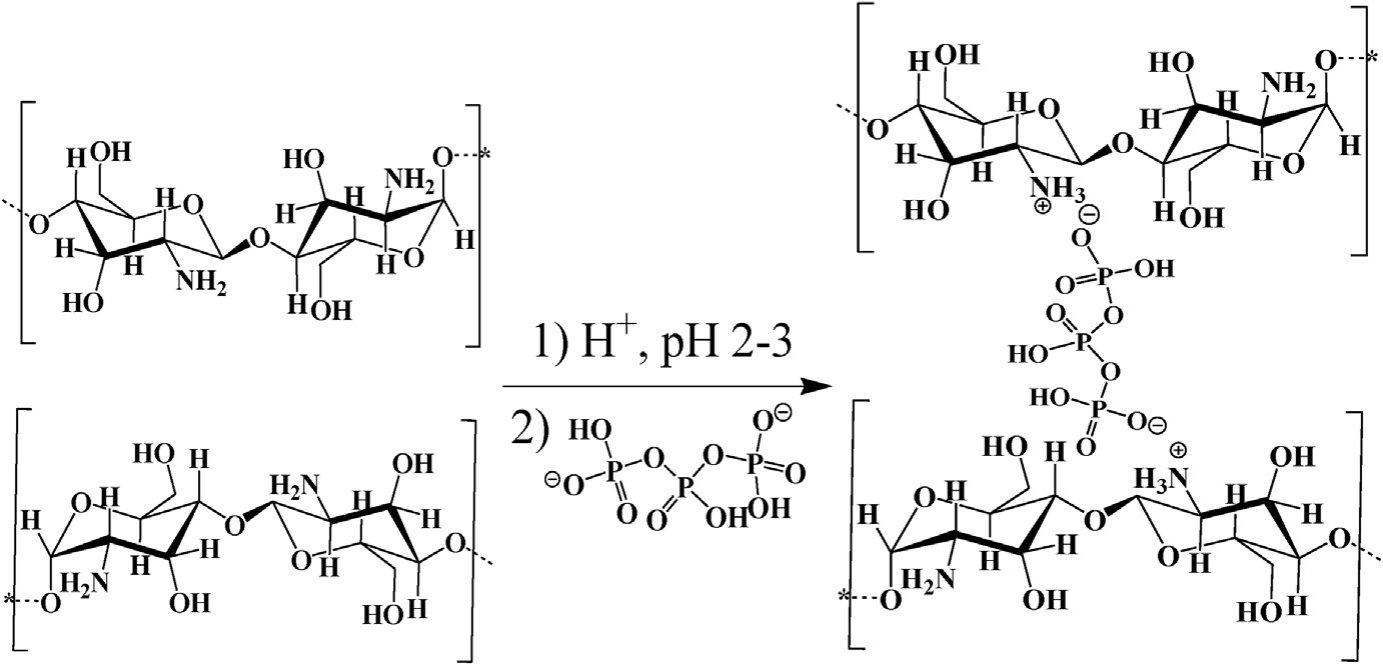
Chitosan ionically crosslinked with TPP.

Polycationic chitosan is soluble in acidic media (p*K*a 6.5) and can interact with negatively charged TPP. The positively charged amino groups of chitosan interact with the negatively charged TPP in a pH-dependent manner and form an intermolecular or intramolecular network structure [38] (Fig. 2).

The crosslinker TPP is generally recognized as safe and permitted to use as a food additive by the U.S. Food and Drug Administration [39].

#### 2.1.3 Chemistry of chitosan crosslinked with glutaraldehyde

**Fig. 3:**
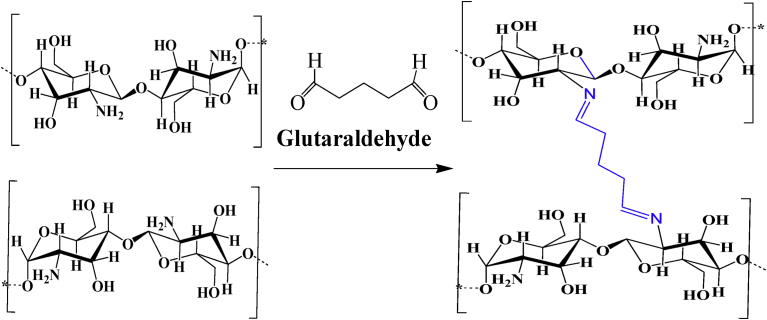
Chitosan chemically crosslinked with glutaraldehyde.

**Fig. 4:**
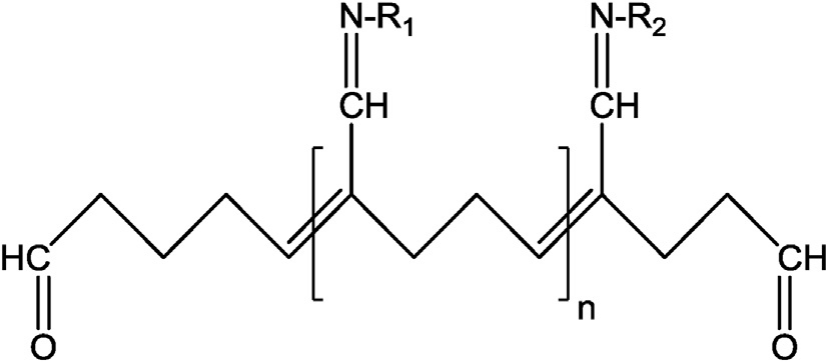
Structure of crosslink formed by Schiff base reaction of glutaraldehyde with amino groups of two chitosan or conjugate repeat units (R_1_ and R_2_). Note the two aldehyde end groups.

**Fig. 5:**
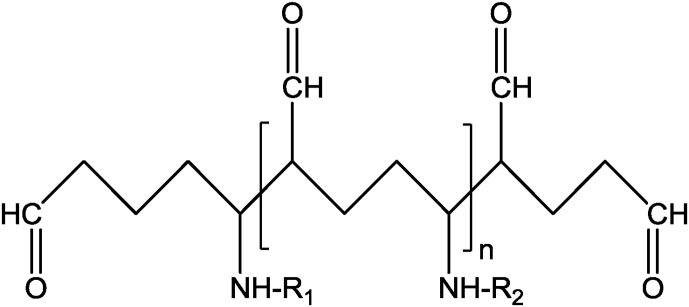
Structure of crosslink formed by Michael-type reaction of glutaraldehyde with amino groups of two chitosan or conjugate repeat units (R_1_ and R_2_). Note the in-chain as well as the two end-chain aldehyde groups.

**Fig. 6:**
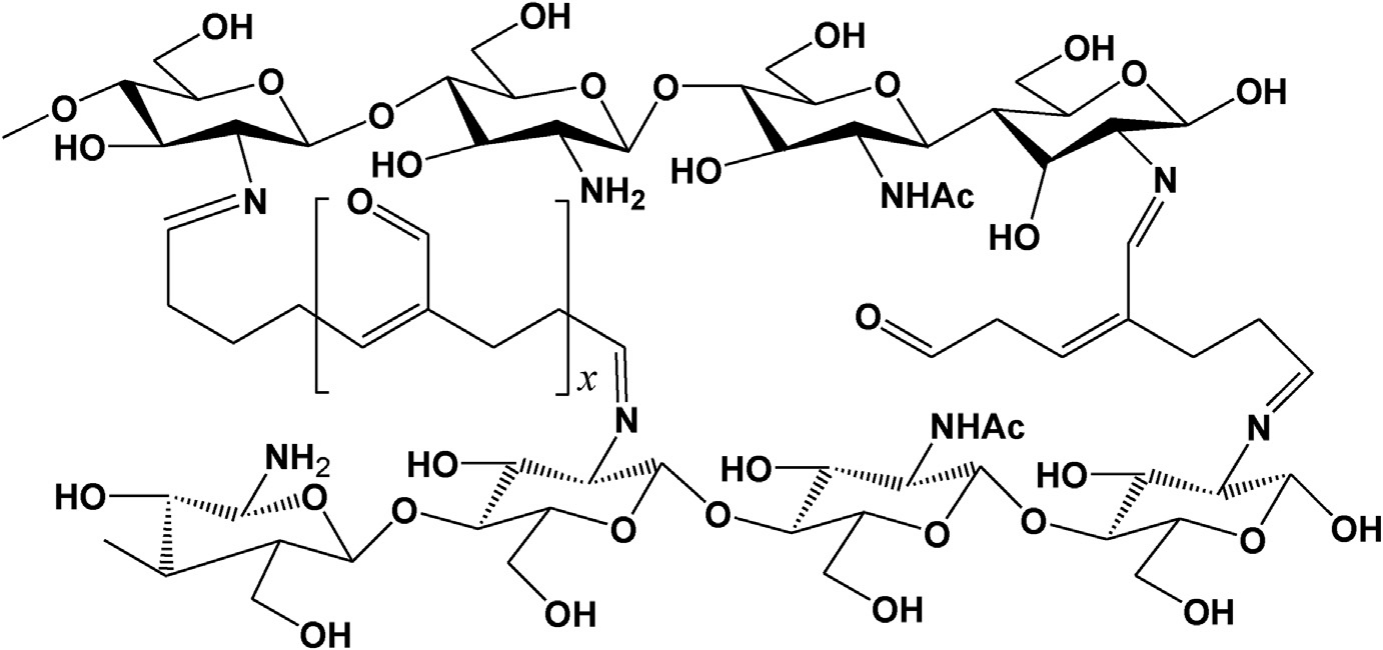
Chitosan catalyses the polymerization of glutaraldehyde and produces inhomogeneous products, Reproduced from [43].

Using glutaraldehyde as a crosslinker, chitosan nanoparticles with or without drug loading has been investigated for drug delivery and medical purposes. Depending on the reaction conditions, i.e., the concentrations of glutaraldehyde and chitosan, and the pH of the reaction medium, the structures of the products are complex and varied. It is assumed that both carbonyl groups of glutaraldehyde are involved in the cross-linking process with chitosan. The structure of the crosslinks formed in glutaraldehyde of amino functional groups produces a covalently bonded product in the chitosan-glutaraldehyde conjugate (Fig. 3) [40]. If the glutaraldehyde crosslinking reaction is assumed to have occurred under acidic conditions to replace all amino groups (-NH_2_) on the chitosan nanoparticle surface with the amine groups, the structure can be shown in Fig. 4 [41]. The other possible crosslink structure that would produce aldehyde groups is shown in Fig. 5 and arises from Michael-type addition rather than Schiff base formation [42]. Depending on the concentration of glutaraldehyde and the pH of the media, chitosan catalyses the polymerization of glutaraldehyde to form inhomogeneous products [43] (Fig. 6), in which the orientation of the bonds between chitosan and glutaraldehyde in nanoparticles would be different and inaccessible to the enzyme to break down the structure.

The crosslinker glutaraldehyde, which is not free from toxicity [44] is known to be a potent irritant, sensitizer and neurotoxic; however, its fate in the human body is not fully understood [45, 46]. Although mechanisms for glutaraldehyde toxicity have been postulated, research on the toxicological, potential of this chemical has shown inconsistent results. In addition, the overexposure of glutaraldehyde to humans produces typical sensory irritant effects on the eye, skin and respiratory tract resulting in chronic dermatitis and asthma. Developmental toxicity studies show glutaraldehyde not to be teratogenic, while percutaneous pharmacokinetic studies showed low skin penetration, with lowest values measured *in vitro* in rats and human skin [45, 47].

#### 2.1.4 Chemistry of chitosan micro/nanoparticles

**Fig. 7:**
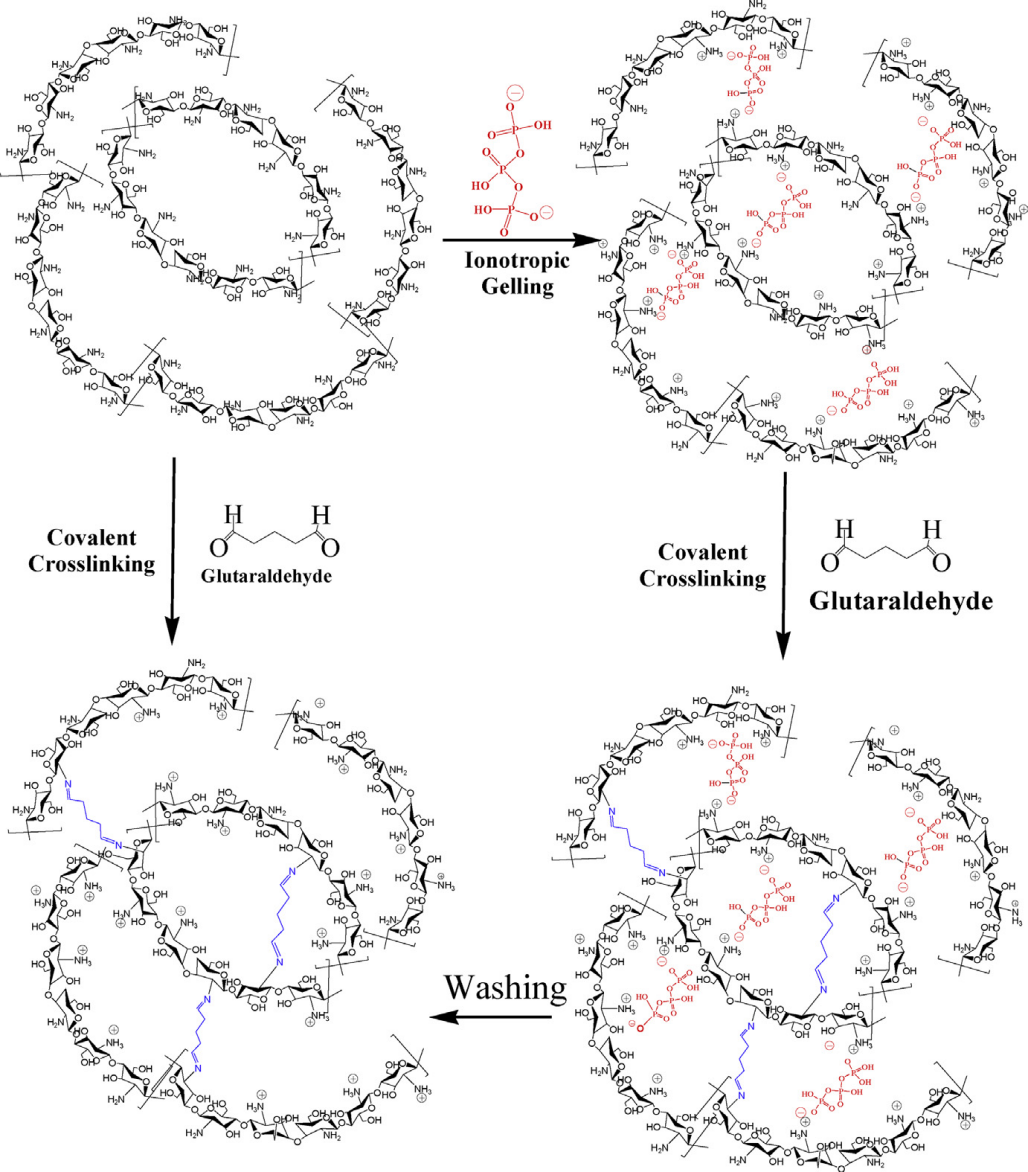
Schematic diagram of chitosan nanoparticles formed by crosslinking agents.

The formation of nanoparticles of chitosan or chitosan crosslinked with either TPP or glutaraldehyde is very complex (Fig. 7). However, it is well established that the inter and intramolecular linkages created between TPP and the positively charged amino groups of chitosan are responsible for the success of the gelation process [48]. Bhumkar and Pokharkar [49]suggested that the mechanism of cross-linking of chitosan with TPP could be either by deprotonation or ionic interaction by adjusting the pH of TPP. In their study, chitosan was cross-linked ionically with TPP at lower pH and by deprotonation mechanism at higher pH.

**Scheme 1:**
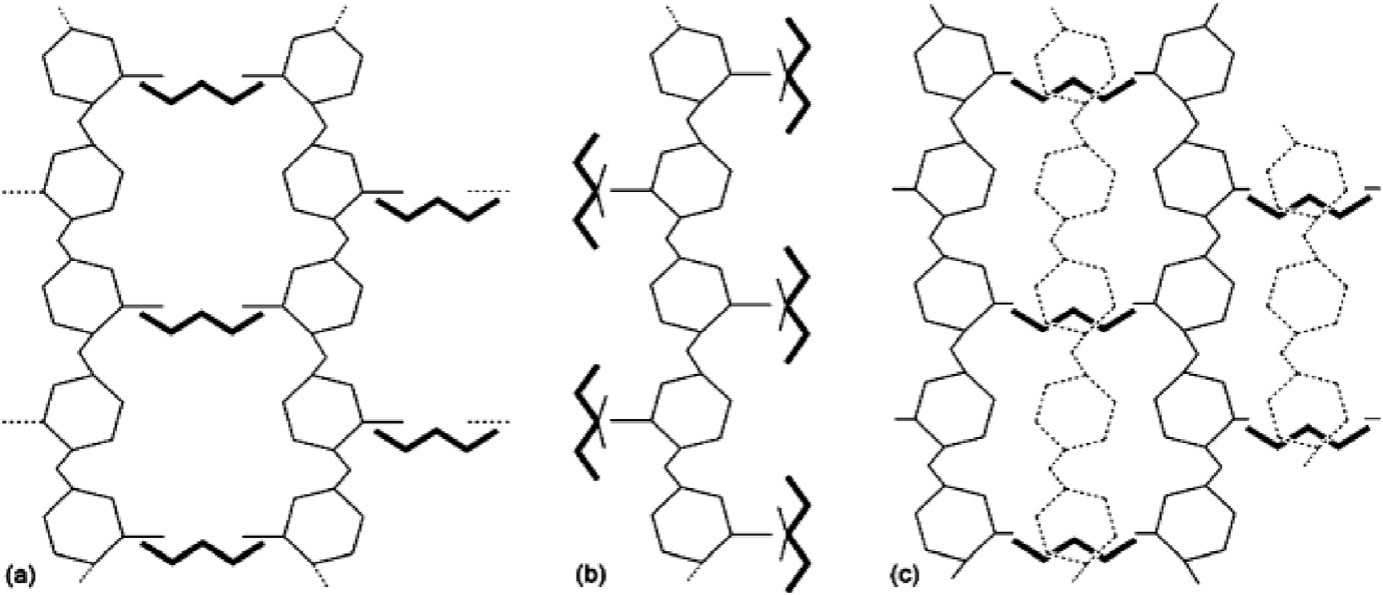
Simplified schematics of the original ionic cross-linking configurations (a) H-link and (b) T-link. Combinations of fundamental linking types lead to (c) secondary linking types. In configuration (c), the dotted monomer structures are off-plane and form T-links with the TPP units below them. Reproduced from [50].

The molecular arrangement of TPP within chitosan-TPP matrices was studied by Koukaras et al [50]using computer-aided molecular modeling based on density functional theory. These studies showed that the most probable cross-linking arrangement of TPP within TPP-chitosan composites is as in Scheme 1.

Due to the formation of complex structures in the nanoparticles, it is unknown whether these structures are degradable (hydrolytic, oxidative or enzymatic) in the biological system or not. To date, no complete in-vitro degradation studies of chitosan micro/nanoparticles have been reported, and no degradation data are available in the biological system. Another critical issue that should be taken into consideration is the stabilization of chitosan-based nanoparticles using a second crosslinking procedure to minimise its burst release effect of the incorporated drug or to enhance storage stability. This procedure is based on introducing a covalent bond following ionic gelation process, and it utilizes carbodiimide coupling or glutaraldehyde, or an oxidation reaction using hydrogen peroxide [30, 51, 52]. Fig. 7 depicts a schematic representation of how a second crosslinking procedure can be involved using glutaraldehyde in nanoparticle stabilisation. Again, the covalent crosslinking involves the formation of a Schiff's base structures (Fig. 4) and/or Michael-type (Fig. 5) products on the surface or within the core of the nanoparticle. However, the spatial distribution of this covalent crosslinking and its impact on the biodegradability of chitosan has not been investigated. As mentioned earlier, the degradation of polymers depends on the reaction (by any mechanisms) between reactants and the vulnerable bonds in the polymer; however, once nanoparticles formed (Fig. 7) due to either ionic bond (i.e., TPP) or the covalent bonds between the polymer and crosslinkers (i.e., glutaraldehyde), the orientation of chitosan structure in particles (currently not known) would be significantly different which could presumably affect the interaction between chitosan and enzyme, which is responsible for chitosan degradation. The question “are chitosan particles really degradable?” posed here is an obvious concern in pharmaceutical and medical applications of polymer micro/nanoparticles.

## 2.2 Mechanism of chitosan degradation

The degradation of polymers occurs by hydrolysis, oxidation and enzymatic reactions [53, 54]. The degradation by hydrolytic mechanism involves the reaction of weak bonds in the polymer with water, and the rate of degradation reaction depends upon the accessibility of water into the polymer matrix rather than the intrinsic rate of ester cleavage [55]. As this review reports the degradation of chitosan, which is generally hydrolysed by enzymes lysozyme and chitinase, we highlight the degradation of chitosan and its micro/nanoparticles by lysozyme, which is available in human body especially in the lungs. The partially N-acetylated derivatives of chitosan were found to be 1.5–4.0 times more digestible than that of N-acetylchitosan, and their enzymatic hydrolysis rate was controlled by the degree of substitution for N-acetyl groups due to the interaction between enzyme and substrate (chitosan) by the hydrophilic free amino groups randomly distributed on the chitosan chain [56]. It has been reported that chitosan molecular weight and it's DA has a direct effect on the biodegradation process since at a greater molecular weight the degradation process is delayed in “*in vitro*” as well as “*in vivo*” [37]. Muzzarelli [57], demonstrated that fully acetylated chitosan is totally insensitive to the enzyme, which is recognized by the presence of at least three consecutive N-acetylated groups. Nwe *et al* [58] investigated the effect of molecular weight and acetylation degree on scaffold used for tissue regeneration. They suggested that lysozyme recognizes N-acetyl glucosamine sequences in the chitin/chitosan molecules; thus lysozyme digestibility increases with increasing degree of N-acetylation in the polymer chain. They reported that the chitosan matrix with high DA broke to pieces of monomers and oligomers of chitosan after a few days of lysozyme treatment and the matrix with low DA remained relatively constant for a long time. The lysozyme degradation rate of a chitosan scaffold is inversely related to the molecular weight and degree of crystallinity of the chitosan and proportionally related to its degree of acetylation, and it was faster in the presence of higher amounts of lysozyme in the degradation medium. The short-term degradation of chitosan with different DA and molecular weights was also studied by Bagheri-Khoulenjani *et al.* [59]. The various grades of chitosan were characterized throughout DA range, molecular weight, crystallinity and swelling ratio. The results revealed that the degradation of high DAs chitosan resulted in no significant changes in molecular weight and DA and this degradation has not occurred through the β-chain scission rather through peptide bond cleavage of acetoamido side groups of the polymer. Chitosan degradation is dependent on variations in the distribution of acetamide groups in the chitosan molecule.

A negligible degradation rate of highly deacetylated (fraction of acetylation0.04) chitosan by lysozyme was also observed [60]. The sulphated derivatives of chitosan have been investigated for degradation by lysozyme, and the derivative of 6-O-sulfate groups was found to be responsible for the high affinity to the sulfated chitosan with lysozyme, whereas the 2-O-sulfate and 3-O-sulfate groups were not favourable [61]. The authors demonstrated that the amount of lysozyme bound to chitosan was very less than those of lysozyme bound with sulphated chitosan. Moreover, the chitosan with a low degree of deacetylation (DD) hydrolysed quickly by lysozyme while chitosan with more 73% DD hardly degraded. Several factors such as the degree of deacetylation, molecular weight, concentrations of polymers and crosslinkers, and environmental conditions (pH and temperature) are associated with the enzymatic degradation of polymers.

Yomota *et al* reported that the enzymatic degradation properties by lysozyme of chitosan films were dependent on the degree of deacetylation of chitosan used and it decreased with an increase in its deacetylation. They also found that the acidic conditions accelerated the degradation compared to the neutral pH. In addition, the type of loaded chemicals greatly affects the drug release in the presence of lysozyme [62]. The *in vitro* and *in vivo* degradation of chitosan-based beads was evaluated by Lim *et al* using solutions of two enzymes that are present in the human body: lysozyme and/or N-acetyl- β-D-glucosaminidase (NAGase). They concluded that there is a sequential degradation reaction of chitosans in the mixture solution of the two enzymes where an initial degradation of chitosan by lysozyme to low-molecular-weight species or oligomers followed by NAGase degradation to monomer forms. NAGase plays a vital role for the full degradation of chitosans in the body, even though NAGase itself can not initiate the degradation of chitosans. The *in vivo* degradation rate of acetylated chitosan beads was faster than the in vitro degradation rate. In addition, the degradation increased with increasing DA of the acetylated chitosans [63]. Muzzarelli [57] reported that, at a greater DA (between 84 and 90%), the degradation process is delayed. Highly deacetylated chitosan (over 85%) shows a low degradation index in the aqueous environment and will degrade after a few months, and a lower DA (between 82 and 65%) would lead to a faster degradation. This feature has an impact on some biological properties in chitosan, such as healing capacity, increase in osteogenesis, and a breakdown process by lysozymes in biological systems [57, 64, 65]. As indicated earlier, chitosan and its nanoparticles crosslinked with glutaraldehyde did not degrade in 0.2 mg/mL lysozyme (available concentration of this enzyme in lung fluids) at 37 °C [21]. It is not clear whether the molecular weight or DDA or both factors are involved in the degradation process and therefore', it is impossible to estimate biodegradation rate of chitosan using the DA alone [36].

## 2.3 Chemistry of lysozyme and interaction with chitosan

Human lysozyme is a protein of 130 residues (Relative Molecular Mass, Mr14693) and belongs to the c-type class of lysozymes and it consists of two domains: an -domain (residues 1–40 and 83–100) and a -domain (residues 41–82). Human lysozyme is a bacteriolytic enzyme that is widely distributed in a variety of tissues and body fluids, including the liver, articular cartilage, plasma, saliva, tears, and milk. It cleaves peptidoglycan in bacterial cell walls by catalyzing the hydrolysis of β-(1,4) linkages between the N-acetylmuramic acid and N-acetylglucosamine groups that occur in the peptidoglycan cell wall structure of certain microorganisms, particularly of Gram-positive bacteria. Lysozyme is highly expressed in hematopoietic cells, and it is found in granulocytes, monocytes, and macrophages as well as in their bone marrow precursors [66]. This enzyme has more degradation activity on chitin than chitosan because chitin has more N-acetyl glucosamine residues. Most of the *in-vitro* studies concluded that the degradation of chitin and chitosan depends on the reducing sugar unit in the degradation medium, the molecular weight of hydrolysates in the degradation medium and the weight loss of chitin and chitosan [67]As indicated earlier, chitosan is a co-polymer of D-glucosamine and N-acetylglucosamine bonded via the β(1-4) linkages, the enzyme lysozyme can hydrolyse the β(1-4) linkages between N-acetylglucosamine and glucosamine in chitosan according to the distribution and proportion of N-acetyl group. The lysozyme only recognizes glycosidic linkages between N-acetylglucosamine units. The interaction of this enzyme is dependent on the degree of N-acetylation and thus more active on chitin rather than on chitosan because chitin has more N-acetyl glucosamine residues. It is well-known that lysozyme can catalyze chitosan hydrolysis, but can't affect chitosan with deacetylation degree higher than 95% [68]. Several studies have investigated lysozyme loaded chitosan nanoparticle to enhance antibacterial activity; however, none of them evaluated the degradation of chitosan during release studies [68, 69, 70].

## 2.4 Degradation studies of chitosan micro/nanoparticles

The degradation studies of polymer nanoparticles are contentious. Almost all researchers used in-vitro degradation system; however, they unanimously claimed that the micro/nanoparticles are biodegradable. The CS nanoparticles prepared with or without crosslinking need to be swelled to accommodate the solvent (enzyme solution) and a significant extent of swelling [71, 72, 73] by the solution is important to create an environment for the enzyme to interact with the polymer for degradation.

Grenha et al. [74] studied the degradation of chitosan (M.W. not known, the degree of deacetylation 86%) NPs crosslinked with TPP incubated in lysozyme solution (0.2 mg/mL; maximum lysozyme concentration in human tracheobronchial secretions) in PBS (pH 7.4) at 37 °C. A significant reduction in NPs size occurred in the first 5 minutes, and the degradation was accelerated at high concentration of enzyme. Recently, Poth et al [75] studied the in-vitro degradation of chitosan (degree and acetylation 17 and 42%)-TPP nanoparticles in lysozyme (1.5 μg/mL) solution at 37 °C and CS(42% DA)-TPP nanoparticles were found to degrade with a particle size reduction of 40% within 4 days; whereas no size changes occurred for CS(17% DA)-TPP nanoparticles even after 7 days. Increasing the lysozyme concentration from 1.5 μg/mL to 150 μg/mL was shown to accelerate the degradation of CS(42% DA)-TPP nanoparticles (particle size reduction of approx.60%) in 7 days. Although significant degradation of CS(42% DA)-TPP nanoparticles occurred, the enzyme concentration of 150 μg/mL was not realistic and no further studies after seven days were carried out. The comprehensive review of Kean and Theanu [76] provides additional information on the degradation of chitosan and its derivatives as well as toxicities of the degradation products.

**Fig. 8:**
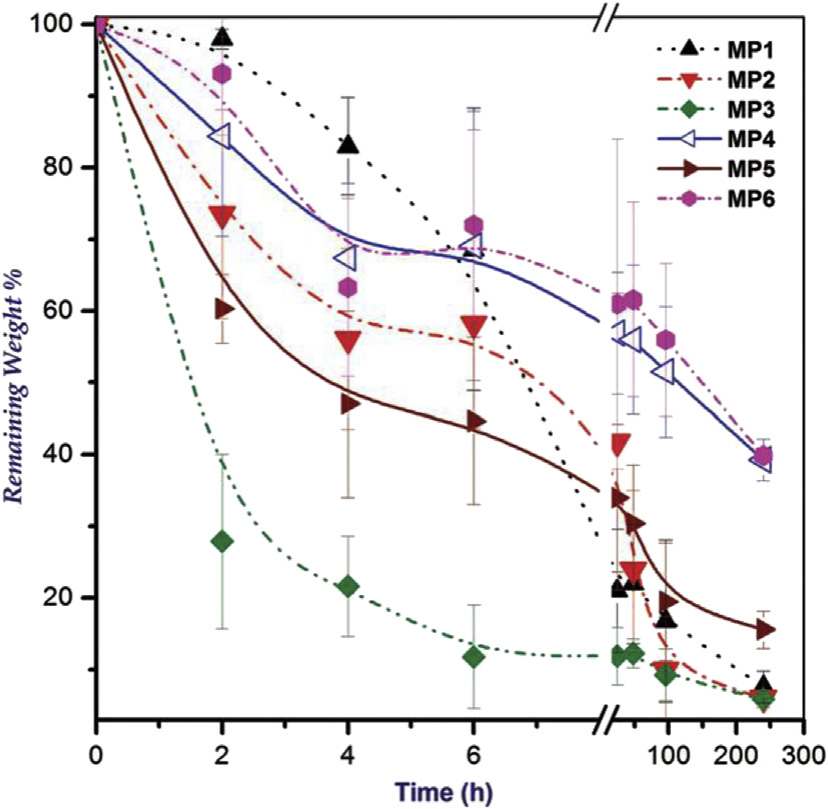
The enzymatic degradation profiles of the prepared chitosan microparticles in phosphate buffer (PBS, pH 7.4) in the presence of lysozyme (adapted from El-Sherbiny and Smyth 2010 [78]).

The degradability of chitosan microparticle prepared by Pickering emulsion photopolymerization technique in 1.0 M NaOH aqueous solution was studied and a 90% weight loss after 35 days was observed [77]. Although an emulsion photopolymerization technique is suitable for preparing chitosan microparticles, the degradation study was conducted in 1M NaOH solution which is not representative of biological fluid, and no complete degradation occurred even in this strong alkaline solution. An in-vitro biodegradation study of PEGylated chitosan microparticles in lysozyme (2 mg/mL in PBS solution 7.4) at 37 °C was carried out by El-Sherbiny et al [78]. The author determined the remaining percentage weight of the microparticles as a function of time and took the outcome as a measure of the enzymatic degradation of particles. The enzymatic degradation started within the first 60 min, and almost half of the mass was lost due to the degradation after 6 h. Interestingly, only 8% mass of the microparticles remained after 240 h, but no studies were carried out to get an idea about the complete degradation (Fig. 8). The authors also emphasized that the presence of high content (40%) of Pluronic reduced the extent of degradation.

Using trimethylated chitosan (TMC) with varying degrees of acetylation (DA), the degradation by lysozyme was found to be highly dependent on the DA of the derivatives, and the highest DA polymers showed the largest decrease in molecular weight. The polymers with a DA <17% (chitosan and derivatives) were found to be less susceptible to enzymatic degradation [79]. The degradation of chitosan was pH dependent as it is not soluble at physiological pH. The authors studied chitosan degradation at pH 4.5 (37 °C) in 38 μg/mL lysozyme solution and marked degradation occurred in the first 24 h; however, no further degradation occurred in 70 h even after applying the fresh lysozyme solution. The authors demonstrated that the chitosan is insoluble at a pH above 6.5 and degradation decreased due to the limited accessibility of the binding sites for lysozyme. Thus, the degradation of chitosan depends upon the molecular weight, degree of deacetylation of polymer, type, and concentration of crosslinkers, concentrations of polymer and enzyme (lysozyme), and pH of the media [17, 73, 75, 79, 80]. The micro/nanoparticles are formed using different crosslinkers, surfactants and grafting agents which are chemically very rigid and are not readily degradable [38].

Most researchers used chitosan or its derivatives with different molecular weights and degree of deacetylations for their degradability studies under different experimental conditions (different chemicals or enzymes, temperatures); however, they used a general term “biodegradable”, although most experiments were not performed in-vivo nor continued until the end to ensure their complete degradation and their elimination or determine their fate in the body. Almost all degradation studies have been undertaken using an in-vitro system and no in-vivo studies have been carried out to fully understand the degradation mechanism. Hou et al [81]investigated the degradation of CS nanoparticles in PBS containing lysozyme. The lysozyme concentration of 100 μg/ml was chosen to better mimic the in vivo physiological conditions. The degradation process was found to be dependent on the concentrations of the enzyme and the crosslinker. The rate of nanoparticle mass loss significantly increased in the presence of lysozyme after four weeks. Approximately 50% of particle weight remained for the CS/TPP nanoparticles prepared at a low TPP concentration in the presence of lysozyme, whereas only 4% of particle mass loss was observed in the absence of lysozyme during the incubation. In addition, a higher crosslinking density reduces penetration and accessibility of the lysozyme to the particle network and subsequently slows degradation. The increase of crosslinker concentrations slowed the rate of gel mass loss, and 80% of particle weight remained after 4-week incubation with lysozyme [81]. In a similar study, the degradability of sunitinib loaded chitosan nanoparticles was evaluated in PBS containing lysozyme at different intervals (24, 48 and 72 h) through the weight loss and shape loss. It was observed that the incorporation of sunitinib into the chitosan nanoparticles had decreased the rate of biodegradability in the presence of lysozyme when compared with bare chitosan nanoparticles. The weight loss was faster at the beginning which can be due to a lower molecular weight part of the nano-polymer dissolving into the degradation medium. Then the fraction of the polymer degraded slowly increased with incubation time due to the bulk hydrolysis [82]. Chin et al [83] evaluated the degradation of BSA loaded glycol chitosan nanoparticles at the highest lysozyme concentration (1.7 mg/mL solution (pH 7.2) compared to physiological conditions. They observed that after 3 h of exposure to lysozyme, drug-free nanoparticles degraded to 10–150 nm particles, whereas BSA-loaded nanoparticles degraded more extensively to predominantly 10–20 nm particles while the protein itself did not fragment [83].

The advancement of nanoparticulate drug delivery is progressing and has achieved remarkable outcomes, but there are many challenges such as the biodegradability and possible toxicity of nanoparticles in biological systems which need to be investigated. Whilst chitosan forms a rigid and complexed structure in nanoparticles as demonstrated in Fig. 7, the orientation of chitosan backbone (especially the β(1-4) linkages between N-acetylglucosamine and glucosamine) would change and thus would impact on the interactions between the largely structured lysozyme enzyme and chitosan units in nanoparticles at physiological pH. Additionally, for chitosan nanoparticles formed by the crosslinkers, i.e., TPP by ionic bonds and glutaraldehyde by covalent bonds, the structure becomes very complex, and the orientation of the β(1-4) linkages would be totally different. Moreover, chitosan catalyses the polymerization of glutaraldehyde and produce inhomogeneous products (as demonstrated in Fig. 5), which might form a different structure in the nanoparticle. Thus, the interactions between the chitosan in nanoparticles and the lysozyme would be inaccessible leading to limit the degradation of micro/nanoparticles. The chitosan complexed with TPP prepared at acidic conditions are transformed into precipitated chitosan chain at physiological fluid (no degradation studies were undertaken) with residual TPP [80]; however, mechanism of breaking the covalent bond between glutaraldehyde and chitosan in physiological condition is not known. Therefore, it is obvious to understand the mechanism of forming chitosan nanoparticles and how lysozyme interacts with the chitosan and breaks the β(1-4) linkages between N-acetylglucosamine and glucosamine in micro/nanoparticles in physiological fluid.

## 3 Conclusions

The application of polymers in drug delivery and biomedical purposes is increasing. Currently, although most of the researchers are advocating the application of chitosan polymer in drug delivery because of their biodegradability and biocompatibility, the reality is far from the claimed application as the complete biodegradability of polymers has not been clearly understood. Based on some in-vitro and very limited in-vivo assays, it is too early to make a decision about biodegradability and biocompatibility of chitosan polymer used in the drug delivery and biomedical applications. The chitosan nanoparticles are formed by the crosslinkers, i.e., TPP by ionic bonds and glutaraldehyde by covalent bonds, respectively. The formed chemical structure becomes very complex (as demonstrated in Fig. 7), and the orientation of the β(1-4) linkages would be totally different, which would affect the interactions between the chitosan in nanoparticles and the lysozyme leading to limit the degradation of micro/nanoparticles. The effect of DA and concentration of the chitosan on the enzyme reaction rate has not been fully understood. Extensive investigations are required to predict the degradation of chitosan nanoparticles crosslinked with either TPP or glutaraldehyde in both *in-vitro*and *in-vivo* systems. More *in-vivo* studies are required to understand the real-life degradation of chitosan, identifications of degradation products and their possible toxicities in the body. We believe chitosan-based nanoparticulate drug delivery will create a promising breakthrough to deliver a large number of therapeutic agents for the management of various diseases and open a new era to use chitosan-based materials for lung drug delivery and other medical purposes in future.

## Declarations

### Author contribution statement

All authors listed have significantly contributed to the development and the writing of this article.

### Funding statement

This research did not receive any specific grant from funding agencies in the public, commercial, or not-for-profit sectors.

### Competing interest statement

The authors declare no conflict of interest.

### Additional information

No additional information is available for this paper.
